# Genetic variants in RET and risk of Hirschsprung's disease in Southeastern Chinese: a haplotype-based analysis

**DOI:** 10.1186/1471-2350-12-32

**Published:** 2011-02-25

**Authors:** Jinfa Tou, Li Wang, Li Liu, Ying Wang, Rong Zhong, Shengyu Duan, Weiguang Liu, Qixing Xiong, Qinglong Gu, Hong Yang, Hui Li

**Affiliations:** 1 Department of Pediatric Surgery, Children's Hospital Zhejiang University School of Medicine, Hangzhou, PR China; 2 Department of Epidemiology and Biostatistics, Institute of Basic Medical Sciences, Chinese Academy of Medical Sciences and Peking Union Medical College, Beijing, PR China; 3 Department of Epidemiology and Biostatistics and MOE Key Lab of Environment and Health, School of Public Health, Tongji Medical College, Huazhong University of Science and Technology, Wuhan, PR China; 4 Department of Otolaryngology, Capital Pediatric Institute, Beijing, PR China; 5 Department of Gastroenterology, Peking Union Medical College Hospital, Chinese Academy of Medical Sciences, Beijing, PR China

## Abstract

**Background:**

Hirschsprung's disease (HSCR) is a classic oligogenic disorder. Except inactivating mutations of RET, some single nucleotide polymorphisms (SNPs) are identified to be associated with the risk of HSCR. This study was conducted to examine the impact of the haplotypes profile of the reported associated SNPs of RET on the risk of HSCR in a Southeastern Chinese population.

**Methods:**

Genotypes of -5G > A (rs10900296), -1A > C (rs10900297), c135G > A (rs1800858), c1296A > G (rs1800860), and c2307T > G (rs1800861) were analyzed in 123 HSCR patients and 168 controls by polymerase chain reaction amplification and direct sequencing. Associations with risk of HSCR were estimated by odds ratio (OR) and their 95% confidence intervals (95% CI) using logistic regression.

**Results:**

We observed a significantly increased risk of HSCR associated with the RET -5AA (OR = 17.75, 95% CI = 7.34-42.92), -1CC (OR = 10.89, 95% CI = 3.13-37.85), 135AA (OR = 13.61, 95% CI = 6.14-30.14), 1296GG (OR = 2.40, 95% CI = 1.38-4.18) or 2307GG (OR = 9.79, 95% CI = 4.28-22.43) respectively. The five SNPs were in strong linkage disequilibrium. The haplotype A-C-A-G-G (OR = 5.06, 95% CI = 1.97-12.99) and diplotype A-C-A-G-G/A-C-A-G-G (OR = 21.08, 95% CI = 5.28-84.09) was also associated with the increased risk of HSCR, indicating a cumulative effect of these SNPs on the susceptibility of HSCR.

**Conclusion:**

These results support the hypothesis that common variations in RET pathway might play an important role in development of HSCR.

## Background

Hirschsprung's disease (HSCR) is a congenital disorder characterized by the absence of enteric ganglion cells in the myenteric (Auerbach's) and submucosal (Meissner's) plexuses of the variable lengths of gastrointestinal tract. The lack of enteric neurons is attributed to the defective proliferation, differentiation, survival, and migration of enteric nervous system (ENS) towards the end of the gut, whereby neural crest cells (NCC) fail to innervate the lower gastrointestinal tract during embryonic development, presenting in clinic with failure to pass meconium, bilious vomiting, enterocolitis-associated diarrhoea, chronic severe constipation and progressive abdominal distension in the neonatal period [1,2]. Based on the length of the aganglionic segment, it can be classified into three subgroups: short-segment aganglionosis (S-HSCR), long-segment aganglionosis (L-HSCR), and total colonic aganglionsis (TCA). Noteworthy, there is significant racial variation in the incidence of the disease worldwide, which is most prevalent in Asians, including China, about 2.8 per 10,000 live births. The male to female ration (M:F) is around 4:1 among S-HSCR patients and 1:1 among L-HSCR patients. Generally, HSCR most commonly presents sporadically although it can be familial and manifests with low, sex-dependent penetrance and phenotypic variability [2].

HSCR is a heterogenic disorder with a number of genes reported to be associated, most of which involved in the signaling pathway implicated in the proper ENS development. RET, a receptor tyrosine kinase, is the major susceptibility gene for HSCR, which locates in 10q11.2 and encodes a transmembrane tyrosine kinase receptor, responsible for triggering a number of downstream signaling transductions [3-5]. There is growing evidence showing that some potentially functional single nucleotide polymorphisms (SNPs) of RET gene could act as low susceptibility factors and modify the phenotype of HSCR, especially in certain combinations of alleles, haplotypes. Specifically, three common SNPs in the coding region of RET, c135G > A in exon2 (rs1800858, A45A), c1296A > G (rs1800860, A432A) in exon7 and c2307T > G (rs1800861, L769L) in exon13, were firstly reported to be associated with HSCR [6-8]. Two promoter polymorphisms -5G > A and -1A > C (rs10900296 and rs10900297) were also reported to be strongly associated with HSCR and have a significantly lower activity in an in vitro dual-luciferase expression assay conducted by Fitze G and his colleagues [2,9]. Garcia-Barceló, *et al *[10] further demonstrated that these two SNPs may disrupt a transcriptional factor, TTF1, binding site and then reduce transcription capacity from the promoter. However, to our best knowledge, looking at these 5 particular SNPs all at once in haplotype profile in the Chinese Han population and their effects on the risk of HSCR have not been fully described.

In this study, we conducted a large case-control study to evaluate the contribution of the genotypes and haplotypes of these aforementioned five SNPs in the pathogenesis of sporadic HSCR in a Southeastern Chinese population.

## Methods

### Study subjects

This case-control study consisted of 123 sporadic HSCR (males: 75.6%) and 194 controls (males: 73.7%). Cases were histological diagnosed with either biopsy or surgical resection material for absence of enteric plexuses in Children's Hospital Affiliated to Zhejiang University Medical School, among which twelve (10%) of patients were affected with long-segment aganglionosis (LSA) and the rest with short-segment aganglionosis (SSA). Controls were normal children taking physical examination in the same hospital during the same time period as the patients were enrolled and frequency matched to cases by sex and age. Both cases and controls were Chinese Han in Zhejiang and informed consent was obtained from their parents. The study was approved by the Institutional Review Board of the Children's Hospital Affiliated to Zhejiang University Medical School.

### Polymorphism Analysis

Genomic DNA was extracted from blood sample of all study subjects by using QIAamp-Blood Kit (Qiagen, Hilden, Germany). Genotypes were determined by PCR and direct sequencing without knowledge of the subjects' case/control status. Fifteen percent of the masked sample was randomly selected and genotyped twice by different people independently. The reproducibility was 100%.

RET SNPs (-5G > A, -1A > C, c135G > A, c1296A > G, and c2307T > G) were genotyped as described previously [6,11,12]. PCR primers for RET -5G > A and -1A > C were 5'-CCCGCACTGAGCTCCTAC-3' and 5'-GGACGTCGCCTTCGCCAT-3', and 5'-TCT CGAGGGATGCTTACTGG-3' and 5'-GGCTCCGGTTAAGGTAGAGG-3' for c135G > A, 5'-CCCTGGTGGGGCATTTGTGC-3' and 5'-GGGTGGTTGGAAACTGATGG-3' for c1296 A > G and 5'-GAACTTGGGCAAGGCGATGC-3' and 5'-ACCCTGCAGCTGGCCTTAC-3' for c2307T > G, respectively. Amplication of the fragments was accomplished under a 25-ul reaction mixture with ~100 ng template DNA, 0.5 uM each primer, 0.2 mM each dNTP, 2.0 mM MgCl_2 _and 1.0 units of *Tag *DNA polymerase with 1 × reaction buffer (Applied Biosystems, Foster City, CA). The PCR profile consisted of an initial melting step of 5 minutes at 95°C, followed by 35 cycles of 40 seconds at 95°C, 30 seconds at 63°C (57°C for c135G > A and c2307T > G), and 45 second at 72°C, and a final elongation step of 7 minutes at 72°C. PCR products were then sequenced using an ABI PRISM^® ^Big Dye™ Terminator v3.1 Cycle sequencing kit (Applied Biosystems, Foster City, CA) and an ABI 3130 automated sequencer according to the manufacturer's instructions.

### Statistical analysis

Chi-square test was used to compare the allele and genotype frequencies between cases and controls, as well as LSA and SSA. The associations between the genotype and the risk of HSCR were estimated by odds ratios (ORs) and their 95% confidence interval (CI), which was calculated by unconditional logistic regression adjusted for gender. PHASE v2.1 [13] was used to estimate the haplotype frequencies composed of five SNP pairs, respectively, and their difference between patients and controls. Diplotype were defined by two haplotypes of each individual. Bonferroni adjustment was used to adjust multiple comparison and p values were considered significantly at a level of 0.003, to allow for the testing of 5 partially correlated markers using 3 different inheritance model (dominant, recessive, and additive model). All of the statistical analyses were performed using Statistical Analysis System software (version 9.12; SAS Institute, Cary, NC).

## Results

### Association of RET SNPs with the risk of HSCR

All the five markers in the controls conformed to HWE (*P *> 0.05, data not shown). The frequency of A allele in -5G > A, C allele in -1A > C, A allele in 135G > A, G allele in 1296A > G and G allele in 2307T > G in cases were 82.5%, 88.6%, 78.9%, 90.7% and 82.1%, which was higher than that of 45.5%, 63.1%, 43.8%, 80.4% and 49.7% in controls, with the *P *for Cochran-Armitage trend test of 1.12 × 10^-16^, 3.43 × 10^-11^, 4.20 × 10^-15^, 5.70 × 10^-4^, and 2.32 × 10^-14^, respectively. All those markers were shown to be strongly associated with the HSCR risk (Table 1). None of these SNPs were significantly different between LSA and SSA patients (data not shown).

**Table 1:** Genotype distribution of the *RET *gene polymorphisms in case-control

Polymorphism	Genotypes	Controls n = 168	cases n = 123	OR*^b ^*(95% CI); *P*
-5G > A, rs10900296 *^a^*	GG	50 (29.8)	7 (5.7)	Reference
	GA	83 (49.4)	29 (23.9)	2.50 (1.02-6.12); 0.046
	AA	35 (20.8)	87 (67.4)	17.75 (7.34-42.92); < 0.001

-1A > C, rs10900297 *^b^*	AA	22 (13.1)	3 (2.4)	Reference
	AC	80 (47.6)	22 (17.9)	2.02 (0.55-7.38); 0.288
	CC	66 (39.3)	98 (79.7)	10.89 (3.13-37.85); < 0.001

c135G > A, rs1800858 *^c^*	GG	52 (31.0)	10 (8.1)	Reference
	GA	85 (50.6)	32 (26.0)	1.95 (0.89-4.32); 0.095
	AA	31 (18.4)	81 (65.9)	13.61 (6.14-30.14); < 0.001

C1296A > G, rs1800860 d	AA	6 (3.6)	0 (0.0)	Reference
	AG	54 (32.1)	23 (18.7)	
	GG	108 (64.3)	100 (81.3)	2.40 (1.38-4.18); 0.002

c2307T > G, rs1800861 *^e^*	TT	37 (22.0)	9 (7.3)	Reference
	TG	95 (56.6)	26 (21.1)	1.08 (0.46-2.53); 0.853
	GG	36 (21.4)	88 (71.6)	9.79 (4.28-22.43); < 0.001

*^a ^p *= 1.12 × 10^-16^, *^b ^p *= 3.43 × 10^-11^, *^c ^p *= 4.20 × 10^-15^, *^d ^p *= 5.70 × 10^-4^, *^e ^p *= 2.32 × 10^-14^, for Cochran-Armitage trend test.

Unconditional logistic regression model was used to estimate RET SNPs effect (additive, dominant, or recessive) according to the smallest AIC (Akaike information criteria) value. Subjects carrying the RET -5AA, -1CC, 135AA or 2307GG genotype had a 17.75-fold (95% CI = 7.34-42.92), 10.89-fold (95% CI = 3.13-37.85), 13.61-fold (95% CI = 6.14-30.14) or 9.79-fold (95% CI = 4.28-22.43) elevated risk for the development of HSCR, compared with the counterpart wild genotype, respectively. The much higher OR value in the present study and previously published association studies indicated that HSCR is a classic oligogenic disorder, which is different from the common complex diseases, such as sporadic cancers, cardiovascular diseases and type 2 diabetes with OR less than 2,. Because the frequencies of wild genotype for 1296A > G were low, we combined it with heterozygous-type genotype together for analysis. Similarly, the homozygous RET 1296GG genotype was also linked to risk of HSCR, with the OR of 2.40 (95% CI = 1.38 - 4.18) compared with the AA or AG genotype.

### RET haplotypes and risk of HSCR

We further analyzed the association of the haplotypes (MAF > 0.05) comprising the five HSCR-associated SNPs with HSCR risk. The haplotype and diplotype frequencies are presented in Table 2 and LD analysis showed that the 5 SNPs are part of the same haplotype block in Chinese (D' > 0.6). The haplotype A-C-A-G-G, was highly associated with an increased risk of HSCR (OR = 5.06, 95% CI = 1.97-12.99, *P *= 0.001). Intriguingly, the haplotype composited with more HSCR-risk allele associated with more elevated risk of HSCR, indicating there is a cumulative effect of these 5 SNPs on the risk of HSCR (*P *= 7.33 × 10^-17^, for Cochran-Armitage trend test). We also investigated the associations between *RET *diplotypes and risk of HSCR. Since the low frequencies of haplotype G-A-G-A-T and G-A-G-G-T, we combined them as reference in the following analysis. In consistent with the results from haplotype, the genetic risk of HSCR was increased in subjects with less copies of the G-A-G-A(G)-T haplotype and more copies of the A-C-A-G-G haplotype, with the *P *values for the Cochran-Armitage test of 3.09 × 10^-15^. Only diplotype with two copy of risk haplotype, A-C-A-G-G/A-C-A-G-G, was found to be associated with increased risk of HSCR (OR = 21.08, 95% CI = 5.28-84.09), when compared with wild type of diplotype, suggesting a haplotype-dosage effect in the genetic susceptibility of HSCR.

**Table 2:** Frequencies and counts of *RET *haplotypes and diplotypes comprising -5G > A, -1A > C, c135G > A, c1296A > G and c2307T > G

Haplotypes *^a^*	Patients (246 chromosomes)		Controls (336 chromosomes)		OR*^b ^*(95% CI); *P*
**(-5; -1; c135; c1296; c2307)**	**%**	**numbers**	**%**	**numbers**	

G-A-G-A-T	2.4	6	6.0	20	reference *^b^*
G-A-G-G-T	8.1	20	25.3	85	0.78 (0.28-2.20);0.641
G-C-G-G-T	1.6	4	8.3	28	0.48 (0.12-1.91); 0.294
A-C-A-A-G	3.3	8	3.6	12	2.22 (0.62-7.97); 0.221
A-C-A-G-T	3.3	8	2.7	9	2.93 (0.78-10.99); 0.111
A-C-A-G-G	72.0	177	34.5	116	5.06 (1.97-12.99); 0.001

**Diplotypes**	**Patients (123 subjects)**		**Controls (168 subjects)**		

G-A-G-A(G)-T/G-A-G-A(G)-T	2.4	3	7.7	13	reference *^c^*
G-A-G-A(G)-T/others *^d^*	4.9	6	19.6	33	0.79 (0.17-3.64);0.761
others/others	3.3	4	11.9	20	0.87 (0.17-4.54);0.869
G-A-G-A(G)-T/A-C-A-G-G	11.4	14	27.4	46	1.30 (0.32-5.23); 0.712
others/A-C-A-G-G	23.6	29	25.0	42	2.98 (0.78-11.42); 0.111
A-C-A-G-G/A-C-A-G-G	54.4	67	8.4	14	21.08 (5.28-84.09); < 0.001

*^a ^*haplotypes with frequencies less than 5% were not shown

*^b ^p *= 7.33 × 10^-17^, *^c ^p *= 3.09 × 10^-15^, for Cochran-Armitage trend test

*^d ^*represented not G-A-G-A(G)-T or A-C-A-G-G haplotypes

## Discussion

This genetic epidemiological study investigated whether genetic polymorphisms in RET, alone or in combination, could have effects on the risk of HSCR in a Southeastern Chinese population. In this case-control study including 123 HSCR cases and 168 age- and sex- frequency-matched controls, we demonstrated that five polymorphisms in RET gene, including two SNPs in the regulatory region and three SNPs in the coding region, had substantial impact on risk of HSCR, separately or collectively. Subjects carrying the homozygous variant genotypes of -5G > A, -1A > C, c135G > A, c1296A > G or c2307T > G SNPs were at an increased risk for developing HSCR. Moreover, we also observed cumulative effects of these five polymorphisms on HSCR risk, demonstrating the haplotype composited with more HSCR-risk alleles rendered the hosts more susceptible to HSCR. To our best knowledge, this is the first study to investigate the contribution of the haplotypes of these five SNPs to the pathogenesis of sporadic HSCR in Chinese population. It lends supports to the previous findings that these SNPs were associated with the risk of HSCR [1,2,7,10,14-16].

Liu CP et al. reported C135 G/A and C1296 A/G at exon 2 and the combination of AG of these two SNPs were associated with susceptibility of HSCR in Southeast Chinese population [17,18]. Also, they observed these two SNPs were in LD with two other coding SNPs at exon 14 and exon 15 in the same population. Our results expanded their findings and found that C135G/A and C1296 A/G were in LD with the two functional SNPs (-5G > A and -1A > C) in the promoter region and C2307T > G in exon 13. Furthermore, we observed 54.4% of the cases but only 8.4% of the controls carried A-C-A-G-G/A-C-A-G-G diplotype, with a more than 21-fold increased risk of HSCR, indicating a cumulative effect of the A-C-A-G-G combination on the susceptibility of HSCR and A-C-A-G-G may represent the core of the susceptibility allele.

Intriguingly, we found that the frequencies of risk alleles of-5G > A, -1A > C, c135G > A, c1296A > G or c2307T > G in Chinese Han were much higher than those in European [19], partially explaining the relatively higher incidence of HSCR in Chinese. In conclusion, we observed a strong association between a haplotype, comprising the 3 most common SNPs in coding region and two in promoter region, and HSCR risk. These results support the hypothesis that common variations in RET pathway play an important role in pathogenesis of HSCR and might provide clues to develop screening and surveillance strategies.

## Conclusion

These results support the hypothesis that common variations in RET pathway might play an important role in development of HSCR.

## Competing interests

The authors declare that they have no competing interests.

## Authors' contributions

JT, LW, LL, YW and RZ designed the study; WL, QX, QG, SD, HY and HL collected blood samples and epidemiological data and performed the experiments; JT and LW performed the statistical analysis and wrote the paper.

## Pre-publication history

The pre-publication history for this paper can be accessed here:

http://www.biomedcentral.com/1471-2350/12/32/prepub
